# Metronomic Chemotherapy for Palliative Treatment of Malignant Oral Tumors in Dogs

**DOI:** 10.3389/fvets.2022.856399

**Published:** 2022-03-31

**Authors:** Nina Milevoj, Ana Nemec, Nataša Tozon

**Affiliations:** Small Animal Clinic, Veterinary Faculty, University of Ljubljana, Ljubljana, Slovenia

**Keywords:** metronomic chemotherapy, cyclophosphamide, malignant oral tumors, dogs, palliative treatment

## Abstract

The aim of this study was to evaluate the efficacy of metronomic chemotherapy in the palliative treatment of various malignant oral tumors in dogs. Our focus was to determine the effect of treatment on local disease control and to assess the tolerability and safety of the treatment in dogs with various oral malignancies. Metronomic chemotherapy with cyclophosphamide was used to treat 12 dogs and was combined with non-steroidal anti-inflammatory drugs in 6/12 (50%) of dogs. A clinical benefit was observed in 6/12 (50%) patients 1 month and in 4/12 (33%) 3 months after treatment initiation. The median survival time of the dogs was 155 days (range 21–529 days). At the end of the observation period, the disease had progressed in 10/12 (83.3%) of the patients. Sterile hemorrhagic cystitis was the most commonly reported side effect of treatment, occurring in 4/12 (33.3%) dogs. The results of our study suggest that metronomic chemotherapy with cyclophosphamide can be, in a subset of dogs, beneficial in the palliation of malignant oral tumors.

## Introduction

Metronomic chemotherapy (MC), also known as low-dose continuous chemotherapy, is characterized by the oral administration of chemotherapeutic agents in low doses on a continuous schedule, without prolonged drug-free breaks (1). This approach was initially designed to overcome the development of resistance to chemotherapeutic agents by inhibiting tumor angiogenesis (2), however, additional mechanisms of action have been uncovered over the past two decades. In addition to its antiangiogenic action, we now know that MC has immunomodulatory effects (3–5), may target cancer stem cells (6–8) and potentially induces a state of tumor dormancy (9, 10).

The veterinary clinical trials that have evaluated the efficacy of MC have mostly been studies in small, heterogeneous cohorts of patients, mostly with advanced disease (11–14). In most clinical trials, cyclophosphamide was used as a chemotherapeutic agent, with or without the addition of a non-steroidal anti-inflammatory drug (NSAID) (15–20). Other agents, such as lomustine (21) and chlorambucil (13, 22) have also been described in the setting of metronomic treatment. When MC alone is used to treat macroscopic tumors, objective response rates are low, with 3–11% partial (PR) or complete response (CR) rates reported (13, 21, 22). However, in those studies, stabilization or minimal disease progression has been noted in 30–67% of cases (13, 21, 22), making MC an attractive option for palliative treatment of tumors. The most common side effect of MC with cyclophosphamide is sterile hemorrhagic cystitis (SHC), which occurs in up to 32% of cases, and the risk of its occurrence increases with longer treatment duration (18, 20, 23, 24). In addition to SHC, mild gastrointestinal and hematological side effects have also been described (17, 18).

The two main pillars of treatment for malignant oral tumors in dogs remain surgery (25) and radiation therapy (26), but alternative treatment options (e.g., electroporation-based treatments) (27) are emerging. To date, there are no studies describing the use of MC in the treatment of oral tumors in veterinary patients. One report describing the use of MC as first-line therapy in the treatment of various tumor types included a dog with oral melanoma that showed a notable response (12), confirming the preclinical evidence for the efficacy of MC in a melanoma-xenograft mouse model (28), while in other studies, patients were not observed to have an observable response (13, 21). Although data on the efficacy of MC for head and neck tumors are also sparse in humans (29, 30), MC could be another treatment alternative for dogs with unresectable oral tumors or for those dogs whose owners decline surgery and/or radiation therapy.

The aim of this retrospective study was to evaluate the role of MC in the palliative treatment of various malignant oral tumors in dogs.

## Methods

### Patient Selection and Medical Records Review

Medical records of dogs with oral tumors, treated with MC between September 2018 and September 2021 at the Small Animal Clinic, Veterinary Faculty, University of Ljubljana, Slovenia, were retrospectively reviewed. The inclusion criteria were as follows: histopathological diagnosis of malignant oral tumor, no previous systemic chemotherapy and at least 30 days follow-up information or information about euthanasia if it was elected <30 days after treatment initiation. In all cases, owners were offered standard treatment (surgery and/or radiotherapy), which they declined. Metronomic chemotherapy was therefore offered as a palliative treatment option.

Data extracted from medical records included age, breed, sex, tumor type and location, clinical stage, previous treatment(s) if any, dose of cyclophosphamide, (neo)adjuvant treatment, tumor dimensions, and tumor volume throughout the treatment period, reported adverse events, treatment duration, follow-up time and cause of death.

### Staging

The initial examination of patients consisted of a complete physical examination with measurement of the tumor in three perpendicular directions (a, b, and c). Tumor volume was calculated using the ellipsoid formula:


$$\begin{array}{l} \text{V}=\text{a}\times \text{b}\times \text{c}\times \pi ÷6 \end{array}$$

The location of the tumor was determined to be in (1) the rostral aspect of the maxilla extending from the level of the first incisor tooth to the level of the second premolar tooth, (2) the rostral aspect of the mandible extending from the level of the first incisor tooth to the level of the second premolar tooth, (3) the caudal aspect of the maxilla caudal to the second premolar tooth, (4) the caudal aspect of the mandible caudal to the second premolar tooth, (5) the mucosa of the oral cavity and oropharynx, excluding the tonsils (31). In addition, tumors were described as either mucosal or gingival (i.e., originating from the dentate jaws). A complete blood count with a differential white blood cell count (Advia 120, Siemens, Munich, Germany) and serum biochemistry (RX-Daytona, Randox, Crumlin, UK) were performed in all patients. The following biochemical parameters were measured initially and at each follow-up examination: blood urea nitrogen (BUN), creatinine, alkaline phosphatase (ALP) and alanine aminotransferase (ALT).

Clinical staging of the disease at the first presentation was performed either by three-view thoracic radiographs (AXIOM Iconos R100, Siemens Healthcare, Erlangen, Germany) or by contrast-enhanced computed tomography (CT) (Somatom Scope, Siemens Healthineers, Erlangen, Germany) of the head, neck and thorax. Blind or ultrasound-guided fine-needle aspiration biopsy of the regional lymph nodes (mandibular and/or retropharyngeal), followed by cytological evaluation was performed whenever approved by the clients, and especially in patients with clinical or radiologic suspicion of metastatic disease. Clinical stage was defined according to the World Health Organization (WHO) criteria for staging of canine oral tumors (32).

### Treatment Protocol and Follow-Up Examinations

All patients received cyclophosphamide (15–25 mg/m^2^/24 h PO) as the MC agent. When NSAIDs were also administered, they were administered for the entire period of MC treatment and piroxicam (0.3 mg/kg/24 h PO) was the agent of choice. In patients who did not tolerate piroxicam, either carprofen (2 mg/kg/12 h PO) or meloxicam (0.1 mg/kg/24 h PO) was prescribed. Follow-up examinations were performed monthly or bimonthly, depending on the patient's condition and the owner's availability.

Physical examination, tumor dimensions (a, b, and c) measurement, complete blood count with a differential white blood cell count (Advia 120, Siemens), and serum biochemistry (RX-Daytona, Randox) were performed at each follow-up visit. Tumors were measured with calipers and the longest diameter of the tumor was used to determine the response to treatment according to the Response Evaluation Criteria in Solid Tumors (RECIST) (33). The data were compared to the measurements obtained on the previous examination. Clinical benefit (CB) was defined as dogs achieving either a complete (CR), partial response (PR) or stable disease (SD); SD had to be maintained for a period of 4 weeks to be classified as such. Treatment side effects were evaluated by interviewing the owners and evaluating laboratory results; they were classified according to the Veterinary Cooperative Oncology Group toxicity scale (VCOG–CTCAE) (34).

### Statistical Analysis

Median survival time (MST) was the primary endpoint of this study. It was calculated using Kaplan-Meier survival analysis, and one dog that was alive at the end of observation period was censored for analysis. Progression-free survival (PFS) was also calculated and defined as the time from treatment initiation to local disease progression (PD), as defined by the RECIST criteria (33). SigmaPlot software (Systat Software, San Jose, CA, USA) was used for graphical representations and statistical analysis.

## Results

### Patients

An initial review of the medical records revealed 24 dogs with oral tumors treated with MC in the selected time period. Six dogs were excluded from the study because the owners refused histopathological examination. Additional five dogs were excluded from the study as they were lost to follow up. One dog was further excluded due to complete excision of the tumor and subsequent clinically non-measurable local disease.

Thus, a total of 12 dogs with oral tumors treated with MC were included in the study (Table 1). In addition to histopathological examination, immunohistochemical staining was also performed in 3/12 (25 %) cases (patients 3, 9, 11) to obtain a definitive diagnosis.

**Table 1:** Patient data and treatment response.

**Patient no**.	**Gender^**a**^**	**Breed**	**Age (years)**	**Tumor type^**b**^**	**Tumor location**	**Clinical stage^**c**^**	**Previous treatment**	**Initial tumor volume (cm^**3**^)**	**CYC^**e**^ dose (mg/m^**2**^/ 24 h)**	**Adjuvant treatment**	**Treatment response** ^**f**^			**PFS^**g**^ (days)**	**Treatment duration (days)**	**Observation time (days)**
											**1 month**	**3 months**	**End of observation period**			
1	F	Borzoi	11	FSA/AM	Caudal maxilla (gingival)	N/A^h^	/	65.4	25	/	PD	PD	PD	30	191	191
2	M	Crossbreed	5	FSA	Caudal maxilla (gingival)	III	/	78.5	16.8	Carprofen (2 mg/kg/12 h)	SD	PR	PD	209	209	313
3	F	American Bulldog	7	SCC/ACA/ high-grade AA	Rostral maxilla (gingival)	III	/	47.71	22.5	/	PD	PD	PD	30	142	142
4	F	West Highland White Terrier	14	SCC/ ameloblastic carcinoma	Caudal mandible (gingival)	III	/	83.73	24.2	/	PD	N/A	PD	30	31	31
5	M	Labrador Retriever	5	SCC	Rostral mandible (gingival)	II	/	5.89	15	Piroxicam (0.3 mg/kg/24 h)	SD	PR	PD	180	240	470
6	F	Cane Corso	8	PCT	Rostral mandible (gingival)	III	/	12.56	21	Piroxicam (0.3 mg/kg/24 h)	SD	PR	PR	339	170	368
7	F	Cocker Spaniel	11	SCC	Mucosa of the oral cavity (mucosal)	N/A	ECT/IL-12 GET^i^	62.8	22.4	/	PD	N/A	PD	30	44	44
8	M	Crossbreed	14	OM	Rostral hard palate (mucosal)	N/A	surgery	2.4	15.5	/	SD	SD	SD	529	529	529^j^
9	F	Cocker Spaniel	10	OM	Caudal maxilla (gingival)	II	/	2.1	15.6	Piroxicam (0.3 mg/kg/24 h)	SD	PD	PD	30	179	179
10	M	Crossbreed	7	CSA	Caudal maxilla (gingival)	III	/	12.0	20	Piroxicam (0.3 mg/kg/24 h)	PD	PD	PD	30	82	155
11	F	Crossbreed	5	OM	Mucosa of the oral cavity (mucosal)	III	/	4.7	25	/	SD	N/A	PD	30	70	70
12	M	French Bulldog	11	OM/NFSA	Caudal maxilla (gingival)	II	ECT/IL-12 GET	42.88	15	Meloxicam (0.1 mg/kg/24h)	N/A	N/A	PD	21	21	21

a *F, female; M, male*.

b *FSA, fibrosarcoma; AM, amelanotic melanoma; SCC, squamous cell carcinoma; ACA, adenocarcinoma; AA, acanthomatous ameloblastoma; PCT, plasma cell tumor; OM, oral melanoma; CSA, chondrosarcoma; NFSA, neurofibrosarcoma*.

c *WHO clinical staging system^d^, Stage I: T_1_N_0_M_0_, Stage II: T_2_N_0_M_0_, Stage III: T_2_N_1_M_0_ or T_3_N_0_M_0_, Stage IV: T_n_N_n_M_1_*.

d *T_1_: tumor ≤ 2 cm in diameter, T_2_: tumor 2–4 cm in diameter, T_3_: tumor >4 cm in diameter; N_0_: no evidence of regional lymph node involvement, N_1_: histologic/cytologic evidence of regional lymph node involvement, N_2_: fixed nodes; M_0_: no evidence of distant metastases, M_1_: evidence of distant metastases*.

e *CYC, cyclophosphamide*.

f *Defined per RECIST criteria (25); CR, complete response; PR, partial response; SD, stable disease; PD, progressive disease*.

g *PFS, progression-free survival*.

h *N/A, not applicable*.

i *ECT/IL-12 GET, electrochemotherapy with IL-12 gene electrotransfer*.

j *Alive at the end of observation period*.

Regional lymph nodes were sampled for cytological examination in 9/12 (75%) dogs; metastases were detected cytologically in 3/9 (33.3%; patients 3, 4, 6). Screening for pulmonary metastases was performed in 11/12 (91.7%) dogs; in 3/11 (27.3%) dogs with three-view radiographs and in 8/11 (72.7%) with CT. No distant metastatic disease was detected in any of these patients. Clinical stage was therefore determined in 9/12 (75%) dogs and ranged from II to III.

Prior treatment was attempted in 3/12 (25%) cases; one dog (patient 8) was treated surgically, and two dogs (patients 7 and 12) were treated with a combination of electrochemotherapy (ECT) and IL-12 gene electrotransfer (IL-12 GET) (27). Patients were included in the study at least 14 days after surgery or 28 days after ECT and IL-12 GET and when disease progression was observed.

### Treatment Results

All dogs received cyclophosphamide as a MC agent. The median treatment duration was 156 days (range 21–529 days) and the median dose administered was 20.5 mg/m^2^/24 h PO (range 15–25 mg/m^2^/24 h PO). NSAIDs were part of the treatment protocol in 6/12 (50%) dogs; 4/6 (66.7%) received piroxicam (0.3 mg/kg/24 h PO), 1/6 (16.7%) received carprofen (2 mg/kg/12 h PO) and 1/6 (16.7%) received meloxicam (0.1 mg/kg/24 h PO). The summary of treatment results is shown in Table 1.

**Figure 1:**
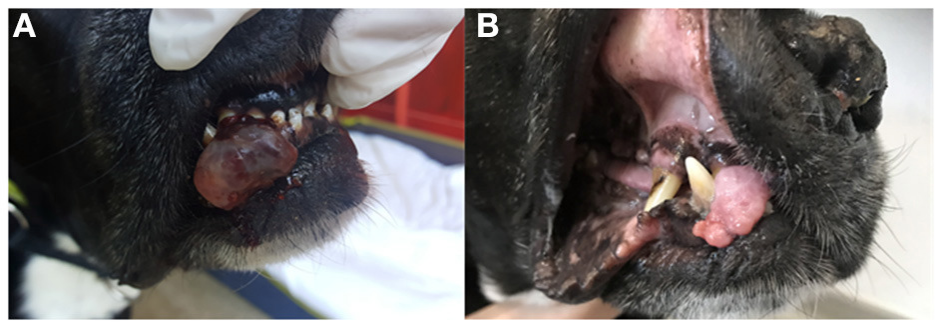
Clinical images of patient no. 6 with an oral plasma cell tumor of the right rostral mandible before MC treatment **(A)** and 2 months after treatment initiation **(B)**. A partial response (PR) was observed after 90 days. The treatment was discontinued 170 days after initiation due to occurrence of sterile hemorrhagic cystitis. The patient was euthanized 368 days after the presentation for presumable tumor-unrelated cause.

**Figure 2:**
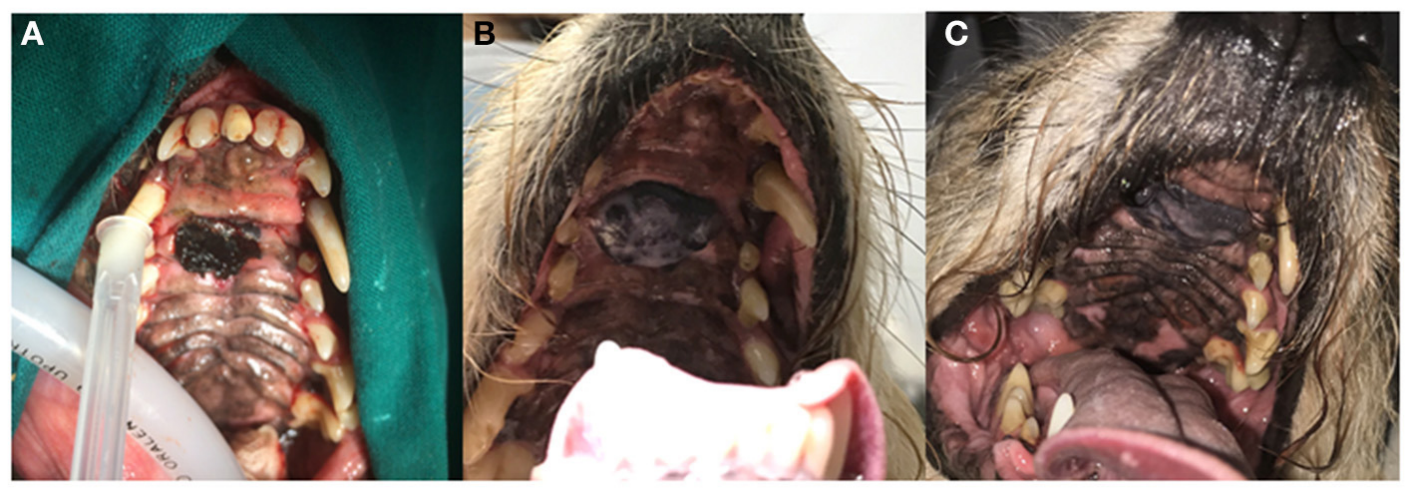
Clinical images of patient no. 8 with an incompletely excised oral melanoma of the hard palate before treatment **(A)**, 270 days **(B)**, and 529 days **(C)** after treatment initiation. Stable disease (SD) was observed. The dog has not experienced any systemic side effects to treatment and was still alive at the time of writing of this report.

Local disease control was assessed at each control examination. Clinical benefit (CB), classified as either PR (Figure 1), or SD (Figure 2), was noted in 6/12 (50%) patients 1 month and in 4/12 (33%) 3 months after treatment initiation. No complete responses (CR) were noted. In the remaining dogs (50%), disease progressed despite treatment. At the end of the observation period (21–529 days, median 167 days), disease had progressed in 10/12 (83.3%) patients. Treatment was permanently discontinued in 4/12 patients (33.3%) due to the onset of SHC.3/4 (75%; patients 2,5 and 6) of those patients experienced CB during treatment.

**Figure 3:**
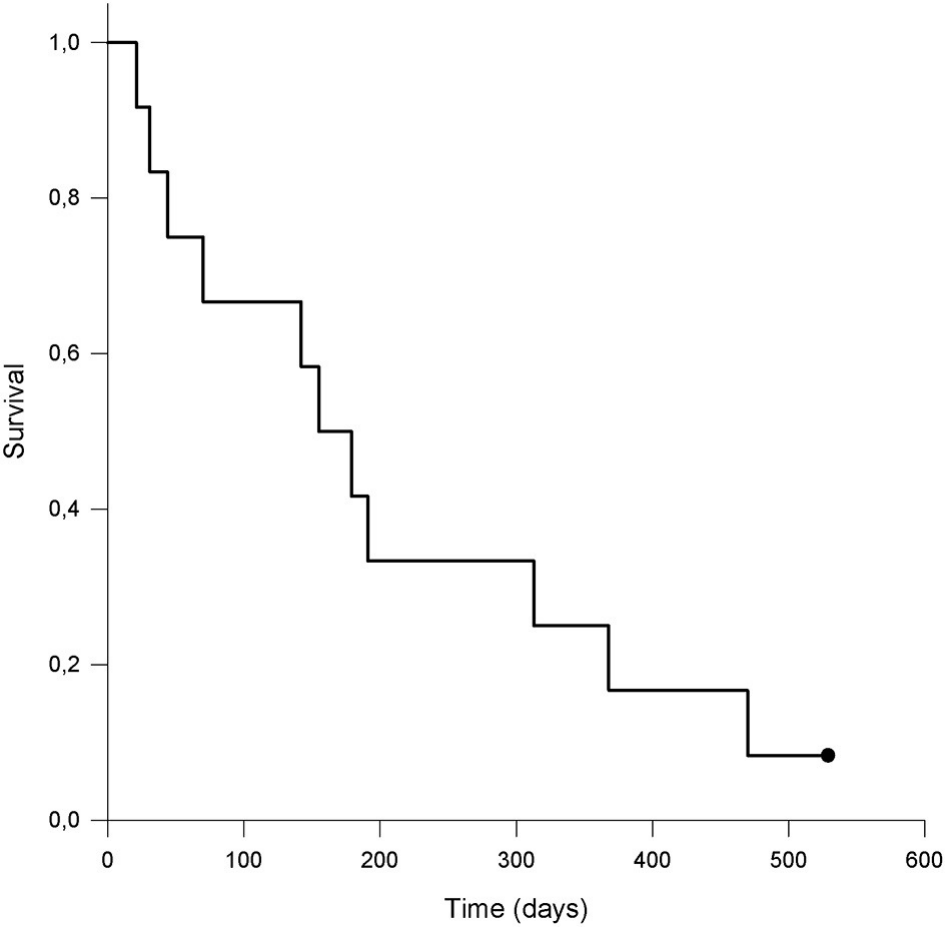
Kaplan-Meier survival curve of dogs (n = 12) with malignant oral tumors treated with metronomic chemotherapy with cyclophosphamide. The dot represents a patient that was alive at the end of observation period and was censored from the analysis. The calculated median survival time (MST) was 155 days.

The calculated MST for all included patients was 155 days (range 21–529 days). 10/12 (83.3%) dogs were euthanized for tumor-related reasons, while 1/12 (8.3%) was still alive and was censored from the analysis. One (8.3%) patient was euthanized for presumable tumor-unrelated reasons; however, a necropsy was not performed to confirm this, so this patient was considered dead secondary to neoplasia and was therefore not censored from the analysis. Kaplan-Meier survival curve is shown in Figure 3. The median PFS was 30 days (range 21–529 days); in dogs that experienced CB, the median PFS was 194.5 days (range 30–529 days). The two dogs that did not develop PD at the end of observation period were censored from the PFS analysis.

### Side Effects of the Treatment

Treatment side effects were classified according to the Veterinary Cooperative Oncology Group toxicity scale (VCOG–CTCAE) (34). Sterile hemorrhagic cystitis (SHC) was the most commonly reported treatment side effect. Grade 1 to 3 SHC occurred in 4/12 (33.3%) dogs 60–170 days after treatment initiation. Metronomic chemotherapy was permanently discontinued in these patients and clinical signs resolved completely in all dogs within 14–72 days after SHC diagnosis. In 3/4 (75%) patients, cyclophosphamide was replaced with metronomic chlorambucil (4 mg/m^2^/24 h PO) and was continued until the time of euthanasia or death.

Apart from SHC, mild (grade 1 to 2) gastrointestinal signs (vomiting and/or diarrhea) were reported by owners in 3/12 (25%) dogs. In these patients, MC was discontinued until the resolution of clinical signs (2–5 days) with supportive treatment (antiemetics, probiotics, gastroprotectants) prescribed as needed. No hematological abnormalities were observed in any of the treated patients.

## Discussion

The results of our retrospective study suggest that MC with cyclophosphamide may be employed as a palliative treatment option for canine malignant oral tumors with a benefit in a subset of dogs. To the authors' knowledge, this is the first study to describe the use of MC for the treatment of various types of malignant oral tumors in dogs.

The incidence of oral tumors in dogs was recently reported to be 0.5%, with the majority of oral tumors being malignant (35). In general, oral tumors, particularly those that are smaller and localized at easily accessible sites, have a good prognosis when surgically removed with clean margins (25). However, in dogs with large tumors or tumors in the caudal oral cavity or palate, adjuvant treatment, such as radiation therapy, is usually required for adequate locoregional disease control. In addition, tumors such as oral melanoma often spread to regional or distant sites, requiring a multimodal treatment approach and still carry a poor prognosis (36). In cases of advanced disease, aggressive and/or expensive treatment(s) may be rejected by the owners and palliative treatment can be used to maintain an acceptable quality of life (37).

Over the past decade, cyclophosphamide-based MC has become increasingly recognized for the treatment of various neoplastic diseases in dogs. Its uniqueness lies in its mode of action; instead of the direct cytotoxic effect observed with maximum tolerated dose (MTD) chemotherapy, MC has a number of mechanisms of action to kill cancer cells. The most important is the antiangiogenic effect, however, it also exerts immunomodulatory effects, may target cancer stem cells, and potentially induce a state of tumor dormancy (38). Metronomic chemotherapy can be used as a first-line treatment or in combination with other anticancer therapies and has beneficial effects on several tumor types, particularly various sarcomas (15–17, 39, 40); however, the beneficial effect of MC was not proven in other studies (41, 42). In a report describing the use of MC with cyclophosphamide to treat 15 dogs with various tumor types, one dog with stage IV oral melanoma was included in the study (12). The dog had responded completely to treatment, was disease-free for 4 months, and subsequently died from a cause unrelated to the tumor (traumatic event). Metronomic chemotherapy with lomustine did, however, not result in an observable response for treatment of two dogs with oral melanoma and two with histologically low-grade, biologically high-grade oral fibrosarcoma (21). Furthermore, metronomic chlorambucil chemotherapy was also not effective for treatment of a dog with an oral carcinoma (13).

In our study, CB during the course of treatment was observed in 50% of patients 1 month and in 4/12 (33%) 3 months after treatment initiation; however, disease progressed in 83.3% at the end of the observation period. A long-lasting response (529 days) with local control was observed in a patient with an oral melanoma that had been surgically pretreated; this was also the only dog that was still alive at the time of writing. This might suggest a potential use of MC either as monotherapy or, in particular, as adjuvant therapy to stabilize the disease and prevent local recurrence or distant progression of the disease in dogs with oral melanoma. Further clinical studies are recommended to elucidate this. Two dogs with rostrally located tumors (patients 5 and 6), experienced a partial response during the course of treatment and also achieved significant survival time (470 and 368 days). These survival times are comparable to previously published survival times of dogs with the same types of tumors treated with standard treatments (31, 43). As cyclophosphamide can be beneficial for treating plasma cell disease in dogs (44), the response and prolonged survival of patient 6 might be attributed to direct cytotoxic effect of cyclophosphamide on the patient's oral plasma cell tumor. In three patients that developed SHC (patients 2, 5, and 10), cyclophosphamide was replaced with chlorambucil after the occurrence of SHC. As CB was observed in patients 2 and 5 during cyclophosphamide treatment, we cannot exclude that the relatively long-lasting responses to treatment could be, at least partially, attributed to chlorambucil.

Sterile hemorrhagic cystitis is the most commonly reported side effect of cyclophosphamide-based MC. This side effect is due to the action of the drug's metabolites, which cause direct injury to the urinary bladder wall (45). The incidence of SHC in our patients was 33.3%, which is consistent with the results of other studies in which the incidence was 7.5–58% (17, 23, 24, 46). It is notable that 3/4 (75%) of dogs that experienced CB discontinued the treatment due to SHC, so the true effect and response to MC might be underestimated in those patients. To reduce the risk of SHC, some authors recommend the concomitant use of low-dose furosemide with MC (24, 45). This approach would be particularly appropriate for dogs receiving long-term MC, since the likelihood of developing SHC increases with the duration of administration and the dose of cyclophosphamide (45). In our study, the diagnosis of SHC was based on the clinical signs of SHC (hematuria, pollakiuria, stranguria) noted by the owners and subsequent urinalysis. To detect the disease at an earlier stage and shorten the time to recovery, urine sediment analysis should be considered at every control visit even in asymptomatic animals. Apart from SHC, mild gastrointestinal side effects were noted by the owners of 25% of the dogs. This is comparable to the results of other studies in which mild gastrointestinal side effects were observed in 14–23.3% of treated dogs (17, 18). However, all dogs that developed adverse effects in our study were concomitantly treated with NSAIDs, which possibly (partially) induced the gastrointestinal side effects. In all patients with gastrointestinal signs, treatment was interrupted for 2–5 days, and the adverse effects resolved either spontaneously or with additional supportive treatment. MC was then continued in the same regime in all dogs. In our study, the evaluation of clinical side effects was based on the reports of the owners and, therefore, might be under- or overestimated. To overcome this, standardized owner questionnaires should be considered in the future.

The present study has some important limitations. The first is the small sample size, which makes it impossible to draw firm conclusions or show differences between the groups of patients with different tumor types or stages. Because of the heterogeneity of the treatment groups, no data were available on the untreated control group. Without a control group, it is questionable whether stabilization of disease in selected patients can be attributed to MC and therefore a definite conclusion can only be drawn in dogs that experienced a PR during treatment. Second, the quality of life of the treated dogs was not assessed with standardized questionnaires, therefore the tolerability of the treatment could only be assessed subjectively by the dogs' owners. The third limitation was the use of WHO classification for staging canine and feline oral tumors, which was published decades ago. Since then, veterinary oncology and diagnostic imaging has greatly evolved and, therefore, this classification might not provide exact clinical stage data. In the future, a revised classification should be implemented, following the example of the revised TNM classification of head and neck tumors in humans (47). However, the staging of oral tumors, and thus the assessment of a patient's clinical stage remains a shortcoming of clinical studies in this field, as there is still no consensus on how to best perform complete staging in dogs with oral tumors (26, 48).

In conclusion, the results of our study suggest that MC with cyclophosphamide may be cost effective in some countries and can be a beneficial palliative treatment in a subset of dogs diagnosed with malignant oral tumors with similar side effects as previously published. Further studies in larger cohorts of patients are warranted to clearly determine the benefit of MC in different types of canine oral tumors including the effect of MC on metastatic disease.

## Data Availability Statement

The raw data supporting the conclusions of this article will be made available by the authors, without undue reservation.

## Ethics Statement

Ethical review and approval was not required for the animal study because metronomic chemotherapy is a standard treatment. It was used in a palliative setting with a written consent of the owner, when standard treatment (surgery and/or radiotherapy) was rejected or unavailable. Written informed consent was obtained from owners for their animals to participate in this study.

## Author Contributions

All authors listed have made a substantial, direct, and intellectual contribution to the work and approved it for publication.

## Funding

This work was supported by Slovenian Research Agency grants P4-0053 and J4-2546.

## Conflict of Interest

The authors declare that the research was conducted in the absence of any commercial or financial relationships that could be construed as a potential conflict of interest.

## Publisher's Note

All claims expressed in this article are solely those of the authors and do not necessarily represent those of their affiliated organizations, or those of the publisher, the editors and the reviewers. Any product that may be evaluated in this article, or claim that may be made by its manufacturer, is not guaranteed or endorsed by the publisher.
